# Synthesis of Zinc Oxide Nanoparticles Using *Rubus fairholmianus* Root Extract and Their Activity against Pathogenic Bacteria

**DOI:** 10.3390/molecules26103029

**Published:** 2021-05-19

**Authors:** Naresh Kumar Rajendran, Blassan P. George, Nicolette N. Houreld, Heidi Abrahamse

**Affiliations:** Laser Research Centre, Faculty of Health Sciences, University of Johannesburg, P.O. Box 17011, Doornfontein, Johannesburg 2028, South Africa; naresh.r84@outlook.com (N.K.R.); nhoureld@uj.ac.za (N.N.H.); habrahamse@uj.ac.za (H.A.)

**Keywords:** zinc oxide, green synthesis, nanoparticles, antibacterial, *Rubus fairholmianus*

## Abstract

Recently, the biosynthesis of zinc oxide nanoparticles (ZnO NPs) from crude extracts and phytochemicals has attracted much attention. Green synthesis of NPs is cost-effective, eco-friendly, and is a promising alternative for chemical synthesis. This study involves ZnO NPs synthesis using *Rubus fairholmianus* root extract (RE) as an efficient reducing agent. The UV spectrum of RE-ZnO NPs exhibited a peak at 357 nm due to intrinsic bandgap absorption and an XRD pattern that matches the ZnO crystal structure (JCPDS card no: 36-1451). The average particle size calculated from the Debye–Scherrer equation is 11.34 nm. SEM analysis showed that the RE-ZnO NPs spherical in shape with clusters (1–100 nm). The antibacterial activity of the NPs was tested against *Staphylococcus aureus* using agar well diffusion, minimum inhibitory concentration, and bacterial growth assay. The *R. fairholmianus* phytochemicals facilitate the synthesis of stable ZnO NPs and showed antibacterial activity.

## 1. Introduction

Metal oxide nanoparticles have received remarkable attention in biomedical technology and are extensively used in engineering and medical applications due to their high surface area. Both the metal and metal oxide nanoparticles hold strong antioxidant and antimicrobial properties, which are widely used for the detection of pathogenic microbes and diagnosis of cancer progression [1]. Metal oxide nanoparticles such as zinc (Zn), calcium (Ca), and magnesium (Mg) oxide nanoparticles at a minimum concentration significantly inhibit microbial growth. ZnO-NPs are commonly used in the production of anti-itch creams, anti-septic lotion, anti-microbial powders, anti-bacterial band-aids, surgical tapes, anti-dandruff lotion, diaper powders, and ceramics [2]. Hamelian and colleagues synthesized silver nanoparticles through a green synthesis method using *Thymus kotschyanus* extract as a reducing agent. The synthesized silver nanoparticles showed strong antioxidant and antibacterial but less cytotoxic effects [3]. Hemmati et al. [4] reported the green synthesis of silver nanoparticles using fritillaria flower plant extract as a reducing and capping agent. Various researchers explained the antimicrobial and antioxidant properties of metal nanoparticles, the metal nanoparticles have shown strong antimicrobial properties, in both in vitro and in vivo experiments [5,6,7,8,9].

Among various nanoparticles, ZnO has recently received much attention due to its unique properties (wide and direct bandgap (3.3 eV) and large excitation binding energy (60 meV)). ZnO is highly catalytic in nature with photochemical activity [10]. Generally, ZnO NPs are produced by several physicochemical approaches such as direct homogenous precipitation, hydrothermal and solvothermal reactions, metal decomposition, chemical vapor decomposition, laser irradiation, mechanochemical milling, and molecular beam epitaxy. The chemical synthesis of ZnO NPs involves the consumption of various organic solvents, oxidizing, and reducing agents that are tedious, expensive, and non-eco-friendly [11]. Hence, there is a need for an alternative approach to synthesize ZnO NPs in an eco-friendly way. In contrast to chemical synthesis, biological nanoparticle synthesis uses microbes, enzymes, fungi, or plants as reducing agents in the production of ZnO NPs [12,13]. Based on previous studies, it is understood that ZnO exhibits significant growth inhibition of a broad spectrum of bacteria [14,15]. Zinc oxide nanoparticles can also be used as a drug delivery vehicle to deliver various drug molecules to the targeted cells [12].

The use of plant and plant-based bioactive compounds for the synthesis of metal nanoparticles is attracting attention due to their excellent reducing capacity and antimicrobial activity and this process is known as green synthesis [15]. In green synthesized nanoparticles, the bioactive/phytochemical compounds present in the plant extract are strongly bound or encased over the surface of nanomaterials that will have both the properties of nanomaterials and phytochemicals. Phytochemicals or plant bioactive compounds, which have strong unique properties of that particular plant (e.g., phenols, vitamins, terpenoids, ketones, aldehydes, and amides), play a pivotal role in treating various diseases. The type of plant and plant extract determines the size and shape of the nanoparticles. The increased biological activity of the green synthesized nanoparticles is due to the synergistic effect of the bioactive compounds present in plants and nanomaterial precursors used for synthesis. The green synthesized nanoparticles have also shown various properties such as electrochemical detection of many antibiotic drugs due to the superior electrochemical performance of biosynthesized nanoparticles [16,17,18].

In recent years, a lot of interest has been raised in isolating plant-based bioactive compounds using alcoholic extracts to perform pharmacological experiments [19]. The main aim of these pharmacological experiments is to control fungal and bacterial infections in human beings. Various ethnomedical plants (e.g., *Alhaji camelorum*, *Anthemis nobilis*, *Berberis integerrima*, and *Zingiber officinale*) are used for their antibacterial and antifungal properties [20,21,22,23]. *R. fairholmianus* is an ethnomedicinally important plant with antioxidant and anticancer properties. Various bioactive compounds were isolated from various *Rubus* species (*Rubus amabilis, Rubus niveus, Rubus sachalinensis, Rubus idaeus, Rubus moluccanus, Rubus ellipticus, Rubus brasiliensis*) and the preliminary phytochemical screening showed the presence of phenolic compounds, anthocyanins, terpenoids, flavonoids, terpenoids, polyphenols, and aldehydes. Studies reported that *R. fairholmianus* root acetone extract showed the presence of many phenolic compounds. Cis-2-(isopentyloxycarbonyl) benzoic acid, 2-(5-methylhexyl) benzoic acid, 4-methylpentyl benzoate, 3-(iminomethyl)-2,4-dimethylphenol, and isopentyl benzoate or 3-methyl benzoate are some of the bioactive compounds isolated from *R. fairholmianus,* which showed strong antioxidant properties, active in inhibiting BRCA oncoproteins and COX inflammatory proteins with in vitro anticancer properties against various cell lines [24,25]. Plant phenolic compounds hold significant antioxidant properties, and antioxidants are well known for their metal ion-reducing properties. This action favors the formation of nanoparticles in the green synthesis method. Moreover, the presence of various proteins, lipids, and amino acids in plants supports the formation of nanoparticles and inhibits nanoparticle cluster formation or particle agglomeration. To date, there are no reports on the use of *R.* *fairholmianus* extracts for the biosynthesis of ZnO NPs. This study is the first report on the synthesis of ZnO NPs through a green chemistry approach using *R. fairholmianus* root extract (RE) as an effective reducing agent, and to evaluate their antimicrobial properties.

## 2. Materials and Methods

### 2.1. Plant Collection, Extraction and Biosynthesis of ZnO NPs

*R. fairholmianus* was collected from Kerala, India, and the authenticity was confirmed (voucher specimen no: BSI/SRC/5/23/2010-11/Tech.1657) by the Botanical Survey of India. The root (100 g) of *R. fairholmianus* was washed under running tap water, dried, and powdered. The powdered roots were extracted with acetone using Soxhlet apparatus. Then, 200 mg of dried extract was dissolved in 10 mL of 0.5% DMSO [25].

Biosynthesis of NPs was carried out according to the procedure of Zheng et al. [26]. Briefly, 10 mL *R. fairholmianus* root extract in 0.5% DMSO was added into 10 mL of 0.5 M zinc nitrate solution and stirred at 80 °C for 48 h. The pale white precipitate formed after centrifugation (30 min at 2500 rpm) was washed with double distilled water. RE-ZnO NPs were collected by drying at 40 °C in a hot air oven. Meanwhile, to compare the biosynthesized NPs, the ZnO NPs were chemically synthesized using sodium hydroxide according to the method of Zheng et al. [26]. A modified green synthesis protocol for the zinc oxide nanoparticles using various plant extracts is given in Table 1.

### 2.2. Characterization of Biosynthesized RE-ZnO NPs

The crystalline structure of RE-ZnO NPs was characterized from 5° to 80° in 2θ by XRD (Panalytical X-PertPro X-Ray Diffractometer with Philips PW1729 diffractometer equipped with Cu Kα radiation source which operates at 45 kV/40 mA). The surface morphology of RE-ZnO NPs was categorized by a TESCAN, VEGA Scanning electron microscope operating at 20 kV and the samples were coated with carbon to acquire higher resolution images. UV-Vis spectrophotometer was used to find the stability of the synthesized RE-ZnO NPs in distilled water and the wavelength used was in the range of 200–800 nm (Schimadzu UV-1208, Kyoto, Japan). Fourier-transform infrared spectroscopy (FTIR) (Perkin–Elmer PE 1600, MA, USA) was used to find out the chemical and functional group of RE and RE-ZnO NPs. For FTIR analysis, the samples were made into pellets using KBr reagent and measured in the spectral range of 400–4000cm^−1^. Finally, thermal properties of RE-ZnO NPs were measured using thermogravimetry (TGA)/differential scanning calorimeter (DSC) (TGA/DSC-60H Schimadzu, Kyoto, Japan) at a heating rate of 10.0 °C/min at room temperature and at 1000 °C in nitrogen gas to establish the ratio of organic/inorganic contents.

### 2.3. Agar Well Diffusion Method for Antimicrobial Activity

The agar well diffusion technique was used to find the antibacterial activity of RE-ZnO NPs using *S. aureus* (ATCC^®^ BAA-1026^TM^). Briefly, a sterile cotton swab was dipped into a broth culture of *S. aureus* (1 × 10^5^ cfu/mL) and spread uniformly on nutrient agar plates. Two agar wells of 5 mm diameter were prepared with the help of a sterilized stainless steel cork borer. About 100 µL of RE-ZnO NPs or RE were added to wells and the plates were incubated at 37 °C for 24 h. Then the zones of inhibition (appearance of clear area/white color area around the wells) and the diameter of each zone of inhibition were measured and the mean values were recorded.

### 2.4. Minimum Inhibitory Concentrations

The minimum inhibitory concentration (MIC) for *S. aureus* (ATCC^®^ BAA-1026^TM^) was determined as sensitivity to the synthesized RE-ZnO NPs, RE, and ampicillin (positive control) using the microdilution assay according to Mandell et al. [32]. Twenty-four-hour fresh cultures were prepared and the standardized inoculum was made and used for the antibacterial assay. In brief, a 96-well plate was prepared by dispensing 190 μL Mueller-Hinton broth (MHB) and 10 μL inoculum (10^5^ CFU/mL) into each well. Various dilutions of RE, RE-ZnO NPs, and ampicillin (1 mg/mL) were mixed with MHB in the microplates containing the previously added inoculums and plates were incubated at 37 °C for 24 h. The well with only MHB served as a blank control. *S. aureus* growth was determined at 590 nm using a microplate reader (Reagen Microplate Reader, NJ, USA).

### 2.5. Bacterial Growth in Different Concentrations of RE-ZnO NPs

*S. aureus* (ATCC^®^ BAA-1026^TM^) suspensions (0.2 mL) were inoculated into corresponding tubes containing 1.5 mL of different concentrations of RE, ZnO NP, and RE-ZnO NPs and 1.5 mL of MHB. To these test tubes, 1 mL of phenol red indicator solution was added. Tubes containing inoculum alone served as positive controls and tubes with RE-ZnO NPs + nutrient media served as negative controls. Test tubes with only MHB served as a blank control and tubes were incubated at 37 °C for 24 h and were observed for change in color and pH.

## 3. Results and Discussion

The successful biosynthesis of RE-ZnO NPs using *R. fairholmianus* root extract was observed by the change in the color of the reaction solution from a brownish-yellow to a pale white after mixing plant extract with zinc nitrate for 48 hr.

**Figure 1:**
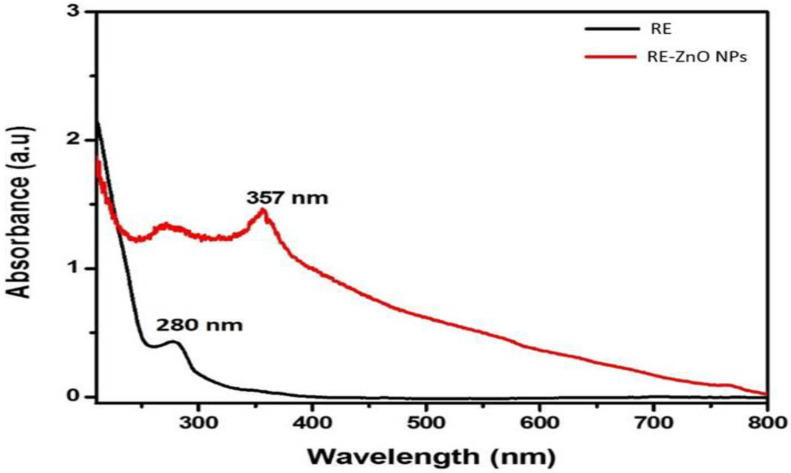
UV-VIS absorption spectra of *R. fairholmianus* root extract (RE) and RE-zinc oxide nanoparticles (RE-ZnO NPs).

Figure 1 displays the UVs absorption spectrum of the RE and RE-ZnO NPs. A peak was observed at 280 nm, which could be attributed to the n–π* transition of the molecules present in the RE. The spectrum of RE-ZnO NPs revealed a peak at 357 nm due to intrinsic bandgap absorption, which confirms the RE-ZnO NPs synthesis. The bandgap energy was calculated at 3.47 eV [33,34].

**Figure 2:**
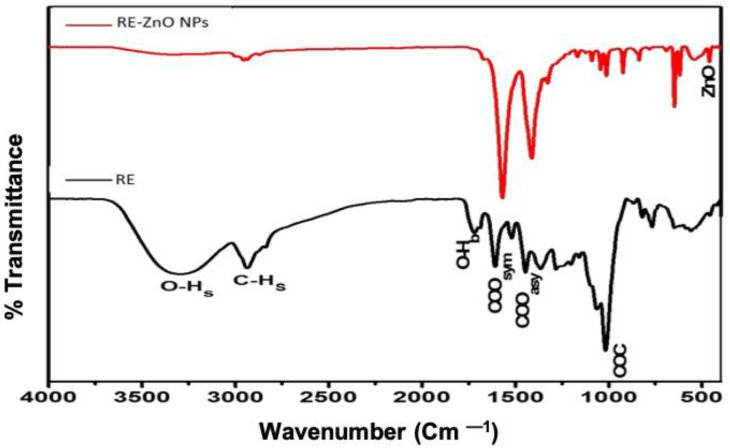
FTIR spectra of *R. fairholmianus* root extract (RE) and RE-zinc oxide nanoparticles (RE-ZnO NPs).

Figure 2 shows the FTIR spectrum of the RE and RE-ZnO NPs. The plant extracts showed various functional peak stretches from 3440 cm^−1^ (hydroxide) to 2856 cm^−1^ (carboxyl) groups. Hydrogen bonds (OH) stretched at 3440 cm^−1^, C-H stretching was observed at 2856 cm^−1^, COO symmetric stretch was found at 1426 cm^−1^, and COO asymmetric stretching at 1626 cm^−1^. Similar types of peaks were also observed in RE-ZnO NPs spectra, which showed a stretching of COO symmetric and asymmetric bonds around 1400–1600 cm^−1^ and indicating the presence of carboxyl groups over the surface of ZnO NPs. The ZnO peak was observed at 486 cm^−1^ and the presence of phytochemical compounds (polyphenolics, flavonoids, tannins, glycosides, saponins, and gallic acids) in *Rubus* (RE) facilitates the formation of ZnO NPs by acting as a reducing and stabilizing agent. Another reason is that the polyphenolic groups present in *Rubus* also promote the reduction of zinc nitrate to zinc oxide and stabilize the formation of RE-ZnO NPs. These spectral results were consistent with the studies of Senthilkumar et al. [34].

**Figure 3:**
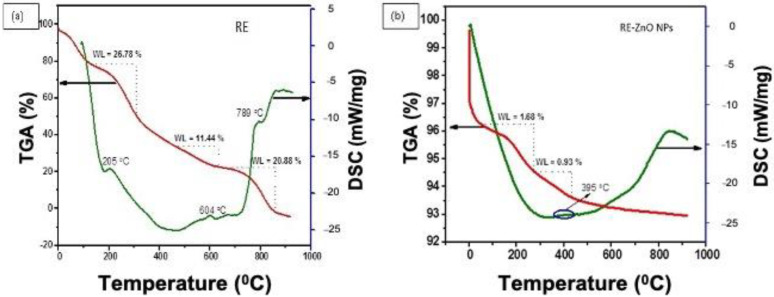
Thermogravimetric analysis spectra of the *R. fairholmianus* root extract (RE) (**a**) and RE-zinc oxide nanoparticles (RE-ZnO NPs) (**b**).

Figure 3a,b describe the TGA/DSC spectra of the RE and RE-ZnO NPs, which displayed heat at 10 °C/Min. TGA was used to decompose the prepared materials, and release water and volatile organic molecules [35,36]. The extract gives three types of weight loss of 26.78%, 11.44%, and 20.88% with corresponding temperatures of 205 °C, 604 °C, and 789 °C, respectively. The weight loss of the extract at 26.78% is due to the removal of water molecules [37]. The weight loss of 11.44% might be due to the decomposition of hydroxide and volatile organic groups [38]. The TGA/DSC spectrum of RE-ZnO NPs is shown in Figure 3b. The DSC curve of RE-ZnO NPs exhibited exothermic and endothermic peaks at 95 °C and 395 °C, respectively. The exothermic peak revealed weight loss (1.68%), which is due to the removal of water and organic volatile molecules from the RE-ZnO NPs. The endothermic peak at 395 °C reveals the decomposition of zinc hydroxide to ZnO NPs with the weight loss (0.93%), as shown in Figure 3b.

**Figure 4:**
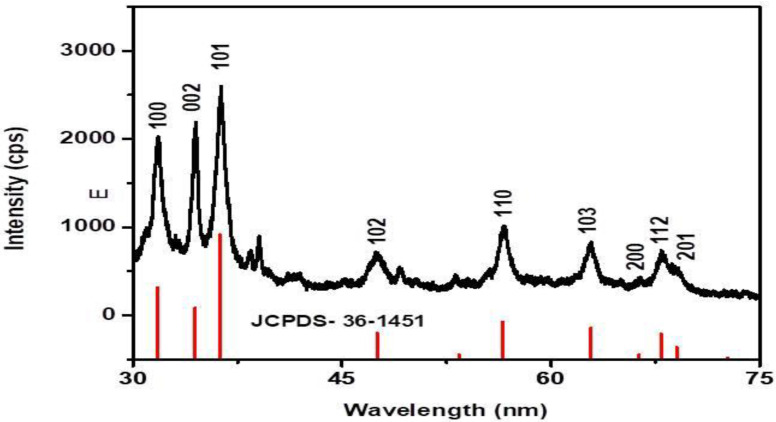
XRD pattern of *R. fairholmianus* root extract (RE) and RE-zinc oxide nanoparticles (RE-ZnO NPs).

Figure 4 describes the XRD pattern of biosynthesized RE-ZnO NPs. The peaks at 2θ 32°, 34°, 36.1°, 46°, 58°, 64°, 66°, 70°, and 72 °C can be indexed to (100), (101), (102), (110), (103), (200), (112), and (201), planes are matching with those patterns present in the International Center of Diffraction Data card (JCPDS card no: 36-1451), which confirms the crystalline nature of the RE-ZnO NPs. The absence of diffraction peaks showed that the synthesized ZnO NPs are pure without any cross-contamination with other molecules and crystalline in nature. The Debye–Scherrer equation was used to determine the size of the RE-ZnO NPs and showed the size is about 11.34 nm. During the biosynthesis process, the aromatic hydroxyl groups (OH) of *Rubus* interact with zinc ions and leads to the formation of RE-ZnO NPs. The unreacted zinc nitrate precursors and intermediate products were observed as a peak around the 30 nm and 60 nm wavelengths. The particle size of the synthesized ZnO NPs was in close agreement with the previous findings of Fakhari et al. who synthesized the ZnO NPs of an average size of 21.49 nm [39].

**Figure 5:**
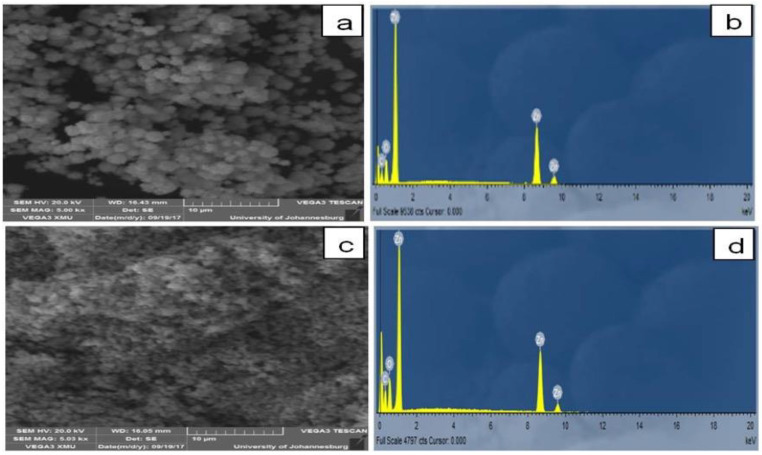
SEM images of RE-zinc oxide nanoparticles (RE-ZnO NPs). (**a**) RE-ZnO NPs; (**b**) Spectrum of RE-ZnO NPs; (**c**) ZnO alone; (**d**) Spectrum of ZnO alone.

Figure 5 displays the SEM image of ZnO NPs synthesized using RE. The excellent distribution of ZnO NPs in RE might be due to the presence of organic compounds in *Rubus*, which facilitates the reduction of zinc nitrate from RE that can offer adequate surface charges between individual ZnO NPs. It was found that the individual particles aggregated together to form larger spherical particles, which were uniformly distributed. The EDAX spectrum shows the presence of Zn and O together with carbon and oxygen, which can be attributed to the extract. The synthesized nanoparticles are aggregated in a spherical shape and showed an average size between 1 and 100 nm. The characterization results obtained in this study were found to be similar to the earlier reports on the biosynthesis of ZnO NPs from algal and other plant extracts [40,41]; however, this is the first report on *R. fairholmianus* mediated green synthesis of ZnO NPs.

**Figure 6:**
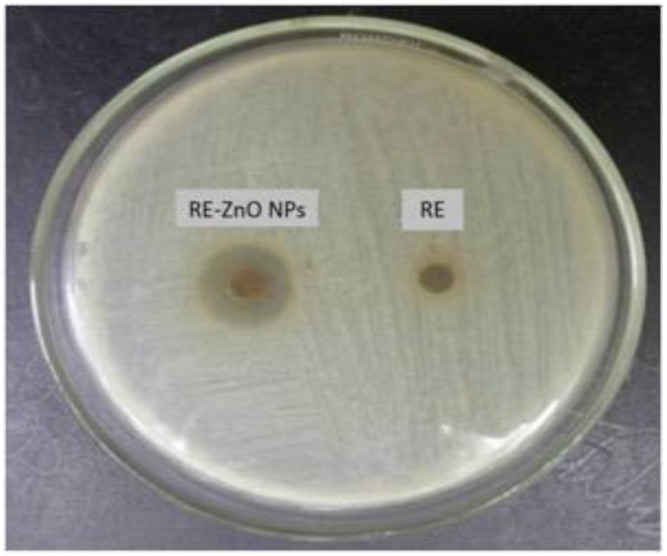
Antibacterial activity of *R. fairholmianus* root extract (RE) and RE-zinc oxide nanoparticles (RE-ZnO NPs) against *S. aureus* in agar well diffusion method.

Table 1 and Table 2 and Figure 6 depict the antibacterial activity of RE, and RE-ZnO NPs against *S. aureus*. The results indicated that RE and RE-ZnO NPs have antibacterial activities at various concentrations against the target bacteria. RE-ZnO NPs displayed the most significant spectrum of activity. The inhibitory effect of RE-ZnO NPs was observed at 157.22 μg/mL (MIC), whereas RE was at 337.86 μg/mL (MIC). Ampicillin showed an inhibitory activity at 0.79 μg/mL (MIC) on *S. aureus.*

Table 3 reveals the antibacterial effect of *R. fairholmianus* root extract (RE), and RE-ZnO NPs at various concentrations. The smaller size of the nanoparticles simplifies their entry through the microbial cell membrane, and thereby inhibits cell growth and promotes bacterial cell death. Metal nanoparticles, mainly ZnO NPs, have strong anti-microbial properties; the mechanism behind this is that ZnO NPs will generate hydrogen peroxides (H_2_O_2_) and these peroxides will disturb the lipid and protein bilayers that lead to the destruction of bacterial cells [42]. The other antimicrobial action of ZnO NPs is that it involves the generation of reactive oxygen species (ROS) and NPs accumulation in the cytoplasm that induces cell death. The generated ROS will damage bacterial proteins, lipids, and DNA to induce cell death [43,44]. Due to the smaller size (1–100 nm), NPs easily breach through the cell wall and enter mitochondria leading to mitochondrial oxidative stress and apoptosis that eventually results in cell death [44]. A recent study by Kumar et al. proved that ZnO NPs prepared with *Raphanus sativus* root extract have excellent antimicrobial activity against MDR strain [45].

Positive (+) = color change (red to yellow) indicating growth of *S. aureus*; Negative (−) = no color change (red) indicating the absence of growth of *S. aureus*.

Previous reports of ZnO NPs from plant extracts showed good antibacterial effects against various pathogenic bacteria. To the best of our knowledge, this is the first study where the *R. fairholmianus* root extract has been used to synthesize ZnO NPs. Plants from the *Rubus* genus were found to possess flavonoids, tannins, and polyphenolics, which could explain their biological properties and strong antibacterial properties. The different phytochemicals in the *R. fairholmianus* extract are responsible for the reduction of zinc nitrate in the formation of RE-ZnO NPs, and its antibacterial activity against *S. aureus*.

## 4. Conclusions

In this study, we reported a green and eco-friendly process to synthesize ZnO NPs using *Rubus fairholmianus* root extract. The initial indication of the formation of ZnO NPs is the change in the color of the reaction solution from a brownish-yellow to a pale white. The size of the biosynthesized RE-ZnO NPs is 100 nm and it is confirmed by SEM analysis. The phytochemicals of *R. fairholmianus* were helpful in the formation of ZnO NPs as evidenced by FTIR results. The XRD study showed that the synthesized RE-ZnO NPs were high in purity, and crystalline in nature. The synthesized RE-ZnO NPs showed strong antimicrobial properties. These findings revealed that *R. fairholmianus* could be potentially used in the production of metal nanoparticles for large-scale synthesis.

## Figures and Tables

**Table 1:** Biosynthetic conditions of nanoparticles synthesized from various plant extracts.

Plant Name	Biosynthesis Conditions	Nature	References
*Mussaenda frondosa* L.	Continuous stirring at 70 °C for 15 min + drying at 400 °C 30 min + calcination at 400 °C	Crystal	[27]
*Cayratia pedata*	Continuous stirring at 65 °C for 20 min + drying at 65 °C for overnight + calcination at 400 °C for 2 h	Fine powder	[28]
*Eucalyptus globulus* Labill	Continuous stirring at 60 °C for 1 h + drying at 100 °C + calcination 400 °C for 2 h	Fine powder	[29]
*Mimosa pudica*	Continuous stirring at room temperature for 4 h + drying at 300 °C for 45 min + calcination 400 °C	Crystal	[30]
*Beta vulgaris*, *Cinnamomum tamala*, *Cinnamomum verum*, *Brassica oleracea* var.	Continuous stirring at 70 °C until white paste formation + calcination 400 °C for 2 h	Coroase powder	[31]

**Table 2:** Minimum inhibitory concentration (MIC) of *R. fairholmianus* root extract (RE) and RE-zinc oxide nanoparticles (RE-ZnO NPs).

*Samples*	Susceptibility (μg/mL)
	*S. aureus*
	MIC
RE	337.86
RE-ZnO NPs	157.22
Ampicillin (positive control)	0.79

**Table 3:** Bacterial growth in different concentrations of *R. fairholmianus* root extract (RE), and RE-ZnO NPs.

Groups	10 μg/mL	20 μg/mL	30 μg/mL	40 μg/mL	50 μg/mL
RE	+	+	+	+	+
RE-ZnO NPs	+	+	-	-	-
Positive Control	+	+	+	+	+
Negative Control	-	-	-	-	-

## Data Availability

Not applicable.

## Sample Availability

Not available.
